# Supplementary material for: Cloud BioLinux: pre-configured and on-demand bioinformatics computing for the genomics community
Source: BMC Bioinformatics. 2012 Mar 19;13:42. doi: 10.1186/1471-2105-13-42 (PMC3372431; doi:10.1186/1471-2105-13-42)
Supplement: Additional file 1 — Supplementary 1 Cloud BioLinux software documentation in the form of a mini, self-contained website. Users need to download and uncompress the .zip file, and open through a web browser the "index.html" file available on the main directory. (ZIP 1823 kb). [file 1471-2105-13-42-S1.ZIP › Cloud-BioLinux-Package-Documentation/docs/velvet.html]

Bio-Linux Software Documentation Pages

Back to search form

## velvet

|  |  |
| --- | --- |
| Name | velvet |
| Description | **Velvet** is a de novo genomic assembler specially designed for short read sequencing technologies, such as Solexa or 454.  Velvet takes short read sequences, removes errors and produces high quality unique contigs. When available, it then uses paired-end read and long read information to retrieve the repeated areas between contigs.  The velvet package is prepared by Debian-med and can be installed on Bio-Linux by typing: `sudo apt-get install velvet` For further details, please see the remote documentation. |
| Homepage | http://www.ebi.ac.uk/~zerbino/velvet/ |
| Remote Documentation | http://www.ebi.ac.uk/~zerbino/velvet/      http://genome.cshlp.org/content/18/5/821.full      http://www.ebi.ac.uk/~zerbino/velvet/Manual.pdf |
